# Sequential pulmonary resections by uniportal video-assisted thoracic surgery for bilateral multiple pulmonary nodules

**DOI:** 10.3389/fonc.2022.961812

**Published:** 2022-10-03

**Authors:** Guangwen Xu, Gaoxiang Wang, Xinyu Mei, Mingsheng Wu, Tian Li, Mingran Xie

**Affiliations:** Department of Thoracic Surgery, The First Affiliated Hospital of University of Science and Technology of China (USTC), Division of Life Sciences and Medicine, University of Science and Technology of China, Hefei, China

**Keywords:** bilateral, uniportal, multiple primary lung cancers, multiple pulmonary nodules, sequential

## Abstract

**Objective:**

The aim of this study was to evaluate the effect of sequential pulmonary resections by uniportal video-assisted thoracoscopic surgery (VATS) for bilateral multiple pulmonary nodules (BMPNs).

**Methods:**

A single-center, prospective, nonrandomized study was performed on patients who underwent one-stage or two-stage operations by uniportal VATS. The clinical, pathological and perioperative data were summarized and analyzed from January 2021 to December 2021.

**Results:**

A total of 80 patients were included during the study period. Sequential pulmonary resection by uniportal VATS was underwent in 40 patients. There were no perioperative deaths and serious complications, 2 patients had postoperative pneumonia, 3 patients had transient atrial fibrillation, 1 patient had persistent severe air leakage, 1 patient occurred hemoptysis. The one-stage group had less operative time, surgical blood loss, pleural drainage, chest tube duration and postoperative admission duration(P<0.05). The results of pathological examination of pulmonary nodules revealed adenocarcinoma *in situ* (n=12), minimally invasive adenocarcinoma (n=24), invasive adenocarcinoma (n=42), squamous carcinoma (n=1),and benign nodules (n=10). The pathological diagnosis included multiple primary lung cancers (30/40, 75%), single primary lung cancer (6/40, 15%). The most advanced pathologic stage of the primary lung cancer was classified as IA (n=19), IB (n=5), II (n=3), and IIIA (n=2).

**Conclusion:**

For patients with excellent pulmonary function, sequential pulmonary resection by uniportal VATS is a safe and feasible for BMPNs. Strict control of surgical indications, reasonable preoperative planning, accurate intraoperative operation, and standardized perioperative management can effectively reduce complications and maximize benefits for suitable patients.

## Introduction

Lung cancer is the leading cause of cancer-related deaths worldwide (1). Owing to the widespread use of low-dose computed tomography (LDCT) screening and advancements in diagnostic technology, the detection rate of bilateral multiple pulmonary nodules (BMPNs) is increasing, among which synchronous multiple primary lung cancers (SMPLCs) account for a large proportion (2, 3). Surgical resection is the main treatment for resectable non-small-cell lung cancer. Video-assisted thoracic surgery (VATS) has become a widely accepted tool for the diagnosis and treatment of thoracic diseases (4). Compared to three-portal VATS, uniportal VATS minimizes the surgical incision, reduces surgical trauma and accelerates recovery (5, 6).

There is no standard treatment for BMPNs requiring resection, and most medical centers currently perform two-stage bilateral pulmonary resection over several months because the perioperative risks related to one-stage surgery are believed to be significantly increased. Currently, data on one-stage surgical strategies are limited, and the safety and effectiveness of sequential pulmonary resection remain controversial.

Herein, we prospectively enrolled patients with BMPNs who underwent one-stage or two-stage operations by uniportal VATS in our department from January 2021 to December 2021. Their clinicopathological and perioperative data were summarized and analyzed to explore the value of sequential pulmonary resection for BMPNs and summarize its perioperative safety.

## Methods

### Patient selection

Patients with BMPNs who were admitted to the First Hospital of the University of Science and Technology of China from January 2021 to December 2021 were enrolled in this study and categorized into two groups according to whether they underwent one-stage or two-stage operations by uniportal VATS. Patients were selected for inclusion based on the following eligibility criteria: (1) surgical indications for the removal of bilateral pulmonary nodules according to the guidelines for the diagnosis and treatment of pulmonary nodules; (2) normal lung function and no surgical contraindications; and (3) achieved R0 resection. Patients were excluded based on the following criteria: (1) abnormal lung function resulting in an inability to tolerate sequential surgery; and (2) refusal to undergo uniportal VATS.

The preoperative staging workup included computed tomography (CT) scanning of the chest and 3D reconstruction of the nodules, abdominal and adrenal ultrasonography, electrocardiography, pulmonary function tests and echocardiography. Patients with solid nodules over 2 cm were examined by magnetic resonance imaging (MRI) of the brain and whole-body bone scintigraphy, and fiberoptic bronchoscopy was performed in patients with central nodules. PET-CT is not a routine examination because of the patient’s economy and nodule size. Routine blood examination was performed before surgery. Patients with invasive adenocarcinoma confirmed by pathological diagnosis were staged according to the AJCC 8th edition TNM staging system (7). Multiple primary lung cancer was diagnosed according to Martini-Melamed criteria (1. Different histology; 2. The same histology but no lymph node metastasis and no extrathoracic metastasis.), and each case was independently staged (8).

This single-center, prospective, nonrandomized study was approved by the Ethics Committee of The First Affiliated Hospital of the University of Science and Technology of China. All patients read and signed an informed consent statement. The vast majority of patients were randomly enrolled, and the patient was informed of the surgical procedure before surgery. However, a small number of patients had a strong desire to choose the surgical method. Due to ethical considerations, the wishes of these patients were respected, and as a result, the patients could not be completely randomized.

### Surgical procedure

The surgical plan was formulated according to the tumor size and location, lung function and intraoperative freezing of bilateral pulmonary nodules. Some nodules were located with a one-time pulmonary nodule localization needle guided by CT before surgery. The priority side of the operation is the side requiring less invasive resection, such as wedge resection before segmentectomy and segmentectomy before lobectomy. If the bilateral excision areas were equal, the right-side operation was performed first.

For one-stage bilateral surgical treatment by VATS, double-lumen endotracheal intubation and single-lung ventilation were performed with the patient in the lateral position on the nonaffected side. An incision was made between the midaxillary line and the anterior axillary line on the fourth or fifth intercostal space. The length of the incision was 3-5 cm(The incision should be based according to bosy shape) (9, 10), and a soft wound protector was used. Thoracoscopy was performed to detect the presence of adhesions, effusion and disseminated nodules in the thoracic cavity and to determine the specific location of the lesion and the anatomy of the lung. Systemic lymph node dissection was performed only if the frozen sections showed invasive cancer. A #26 drainage tube was indwelled at the operation hole to connect the water-sealed bottle or silicone rubber drainage tube to the connection ball. After the one-sided procedure was complete, the patient was rotated to the opposite side for the second resection.

For two-stage bilateral surgical treatment by VATS, the anesthesia and operative steps were the same as those used for one-stage VATS. The interval between the primary and secondary surgery was at least 1 month.

For both groups, the chest tube was removed according to the following criteria: (1) the daily chest drainage volume was less than 200 mL without air leakage, and (2) no pneumothorax or localized pleural effusion was observed on chest X-rays.

### Controlled and observed variables

The clinical data included age, sex, smoking history, family history of cancer, preoperative complications and pulmonary function. The perioperative indicators included operative time, surgical blood loss, pleural drainage, chest tube duration, postoperative admission duration, postoperative complications and surgical procedures. The pathological data included pathological type, TNM staging and pathological diagnosis.

### Statistical analysis

Statistical analyses were performed with SPSS 19.0 software (SPSS Inc., Chicago, IL, USA). Normally distributed data are shown as the means ± standard deviation. Independent samples t tests were used to compare groups, χ2 tests were used to compare two groups, and the Mann–Whitney test was utilized for non-normally distributed measurement data. All P values were two-sided, with statistical significance evaluated at the level of 0.05.

## Results

### Patient characteristics

In total, 80 patients were included during the study period. A total of 40 patients (12 men and 28 women; mean age 52.78 ± 9.30 years) were included in the one-stage group, and 40 patients (17 men and 23 women; mean age 56.87 ± 11.081 years) were included in the two-stage group. All patients had normal pulmonary function, and there was no significant difference with respect to FEV1 (P=0.185) or FEV1% Pred between the two groups (P=0.078). Comparisons of the general clinical data between the two groups, including age, sex, smoking history, family history of cancer and preoperative complications, revealed no significant differences (P > 0.05) (**Table 1**).

**Table 1:** Comparison of patients’ clinical characteristics of the two groups.

Characteristics	One-stage (n = 40)	Two-stage (n = 40)	P
Age (mean, years)	52.78 ± 9.30	56.87 ± 11.08	0.078
Sex Male Female	12 28	17 23	0.245
Smoking history Yes No	4 36	6 34	0.499
Family history of cancer Yes No	3 37	5 35	0.456
Preoperative complications Hypertension Diabetes Arrhythmia Cerebrovascular disease	2 3 3 2	4 2 4 2	0.617 0.671 1.000 1.000 1.000
Pulmonary function FEV1 (mean, L) FEV1% Pred (mean, %)	2.11 ± 0.41 98.73 ± 5.23	1.99 ± 0.44 98.45 ± 5.73	0.185 0.078

FEV1,preoperative-forced expiratory volume in 1 s; FEV1% pred, FEV1%/ (mean FEV1%).

### Perioperative outcomes

No perioperative deaths or serious complications occurred. Lobar-sublobar resection was performed in 30/40 patients (75%), including lobectomy and segmentectomy (n=11), lobectomy and wedge resection (n=16), and lobectomy and multiple sublobar resection (segmentectomy and wedge resection) (n=3). Sublobar-sublobar resection was performed in 10/40 patients (25%), including segmentectomy and segmentectomy (n=6) and multiple wedge resection (n=4). No statistically significant differences were observed between the two groups in terms of postoperative complications or the types of surgical procedures performed (P > 0.05).

Compared to the two-stage group, the one-stage group had a shorter operative time (148.42 ± 49.24 vs. 197.558 ± 74.60 min, P=0.001), less surgical blood loss (29.21 ± 19.07 vs. 58.60 ± 12.98 ml, P<0.001), less pleural drainage (375.95 ± 243.69 vs. 375.95 ± 243.69 ml, P=0.003), a shorter chest tube duration (3.38 ± 1.49 vs. 7.38 ± 4.60 days, P<0.001), and shorter postoperative hospital stay (4.08 ± 1.81 vs. 8.38 ± 4.66 days, P<0.001) (**Table 2**).

**Table 2:** Comparison of perioperative clinical data of the two groups.

Characteristic	One-stage (n = 40)	Two-stage (n = 40)	P
Operative time (mean, min)	148.42 ± 49.24	197.58 ± 74.60	0.001
Surgical blood loss (mean, ml)	29.21 ± 19.07	58.60 ± 12.98	< 0.001
Pleural drainage (mean, ml)	375.95 ± 243.69	642.25 ± 498.35	0.003
Chest tube duration (mean, days)	3.38 ± 1.49	7.38 ± 4.60	< 0.001
Postoperative hospital stay (mean, days)	4.08 ± 1.81	8.38 ± 4.66	< 0.001
Postoperative complications Pulmonary air leakage Hemoptysis Pneumonia Arrhythmia	1 1 2 3	2 0 1 4	1.000 1.000 1.000 1.000 1.000
Surgical procedures Lobar-lobar Lobar-sublobar Sublobar-sublobar	0 30 10	1 29 10	1.000

Operative time, Surgical blood loss, Pleural drainage, Chest tube duration and Postoperative hospital stay was the sum of perioperative indicators of the first operation and the second operation.

Compared with the perioperative outcomes of the first operation and the second operation in the two-stage group and the one-stage group, it was found that the operation time of the one-stage group was longer(148.42 ± 49.24 vs.113.75 ± 52.71 min, P=0.003; 148.42 ± 49.24 vs.83.58 ± 43.61 min, P < 0.001), and the difference was statistically significant. However, there was no statistical difference in the surgical blood loss, pleural drainage, chest tube duration and postoperative hospital stay (**Table 3**).

**Table 3:** Comparison of perioperative clinical data of the two groups.

Characteristic	One-stage (n = 40)	Two-stage (n = 40)	P
Operative time (mean, min)	148.42 ± 49.24	113.75 ± 52.71 83.58 ± 43.61	0.003 < 0.001
Surgical blood loss (mean, ml)	29.21 ± 19.07	25.63 ± 12.57 26.53 ± 21.21	0.165 0.343
Pleural drainage (mean, ml)	375.95 ± 243.69	330.88 ± 246.79 311.38 ± 262.05	0.414 0.260
Chest tube duration (mean, days)	3.38 ± 1.49	3.80 ± 2.27 3.58 ± 2.37	0.327 0.654
Postoperative hospital stay (mean, days)	4.08 ± 1.81	4.33 ± 2.27 4.05 ± 2.43	0.589 0.959

Two-stage: The above is the perioperative results of the first operation, and the following is the perioperative results of the second operation.

### Pathological features

Among the patients undergoing the one-stage procedure, a total of 89 pulmonary nodules were removed from 40 patients, including 2 pulmonary nodules in 31 patients and more than 2 pulmonary nodules in 9 patients. The results of pathological examination of the pulmonary nodules revealed adenocarcinoma *in situ* (AIS, n=12), minimally invasive adenocarcinoma (MIA, n=24), invasive adenocarcinoma (n=42), squamous carcinoma (n=1), and benign nodules (n=10). The pathological diagnosis of these 40 patients included SMPLCs (30/40, 75%), single primary lung cancer (6/40, 15%), and bilateral benign nodules (4/40, 10%). The most advanced pathologic stage of the primary lung cancer lesion in each patient was classified as IA (n=19), IB (n=5), II (n=3), and IIIA (n=2).

No statistically significant differences were observed between the two groups in terms of pathological type, TNM stage, pathological diagnosis or location (P>0.05) (**Table 4**).

**Table 4:** Comparison of patients’ pathological characteristics of the two groups.

Characteristic	One-stage (n = 40)	Two-stage (n = 40)	P
Pathological type Adenocarcinoma Squamous carcinoma MIA AIS Benign nodules	42 1 24 12 10	52 3 14 5 9	0.115
TNM stage 0 IA IB II IIIA	9 19 5 3 2	7 16 8 3 5	0.891
Pathological diagnosis SMPLCs Single primary lung cancer Bilateral benign nodules	30 6 4	32 6 2	0.689
Location RUL RML RLL LUL LLL	25 5 17 31 11	27 6 9 24 15	0.438
Size (mean, mm)	10.66 ± 3.36	10.57 ± 3.43	0.852

AIS, adenocarcinoma in situ; MIA, minimally invasive adenocarcinoma; SMPLCs, synchronous multiple primary lung cancers; RUL, right upper lobe; RML, right middle lobe; RLL, right lower lobe; LUL, left upper lobe; LLL, left lower lobe.

## Discussion

Low-dose computed tomography (LDCT) has gradually become an important means of early screening and diagnosis of lung cancer, and ground glass opacities (GGOs) and ground glass nodules (GGNs) have become typical imaging findings (3, 11). The clinical features of GGOs can indicate malignant tumors or benign disease, such as focal interstitial fibrosis, inflammation, bleeding and adenocarcinoma (12). However, most early lung cancer cases exhibit GGOs on imaging (13). The sensitivity and specificity of positron emission tomography/computed tomography (PET/CT) for the diagnosis of pulmonary nodules are both approximately 80%, but the false positive and false negative rates are high, especially in patients with tumor diameters less than 1 cm (14). CT-guided percutaneous pulmonary puncture is one of the methods used to obtain a pathological diagnosis, but the false negative rate is high because of the small amount of tissue sampled. The gold standard for the differential diagnosis of benign and malignant pulmonary nodules is pathological diagnosis after surgical resection. In this study, 36 cases of lung cancer were confirmed by pathology, with a diagnostic rate of 90%, with 75% being SMPLC and 15% being single primary lung cancer. A total of 89 pulmonary nodules were removed, of which 79 were malignant, representing a malignancy rate of 88.76%. The incidence of multiple nodules in patients with lung cancer is approximately 0.2%-20%, which is consistent with the results of this study (15). MPLC and intrapulmonary metastatic cancer both exhibit multiple pulmonary nodules. However, there are significant differences in the treatment approach and long-term prognosis of multiple primary lung cancer and intrapulmonary metastatic cancer. Therefore, clinicians must accurately judge the nature of multiple pulmonary nodules to avoid misdiagnosis. Based on our experience, we chose to analyze and compare the imaging and management strategies recommended the by ACCP guidelines, Asian consensus guidelines, Fleischner Association guidelines, and the Chinese expert consensus for the diagnosis and treatment of pulmonary nodules and NCCN clinical practice guidelines for non-small-cell lung cancer (16–19). Round-like nodules with a peripheral distribution, morphological isolation and mixed density on imaging can be accompanied by burr lobulation and bronchial vacuole signs. Patients exhibiting the characteristics of pleural traction or vascular bundles should be closely followed up, and the timing of the operation should be optimized.

Compared to thoracotomy, VATS has several advantages, such as significant reductions in pain, the recovery time and the incidence of complications, and can confer better postoperative quality of life to patients (5, 6). Migliore et al. reported the single port VATS technology for the first time (19). Rocco et al. reported the use of uniportal VATS for the diagnosis and treatment of thoracic diseases in 2004 (20). Since then, the indications for uniportal VATS have expanded. Uniportal VATS can be used to complete all types of lung resections and minimizes the surgical incision, further reducing surgical trauma and accelerating recovery (21, 22). Due to recent technological advancement, an increasing number of surgical methods for early lung cancer have been developed, from lobectomy to anatomical segmentomy (23). Segmentomy not only removes the lesion completely but also allows for the retention of more lung tissue. If bilateral lung surgery is completed at the same time, uniportal VATS is indirectly minimally invasive. In this study, lobar-sublobar resection was performed in 30 patients, and sublobar-sublobar resection was performed in 10 patients. Compared to the two-stage group, the one-stage group had a shorter operative time (148.42 ± 49.24 vs. 197.558 ± 74.60 min, P=0.001) and less surgical blood loss (29.21 ± 19.07 vs. 58.60 ± 12.98 ml, P<0.001). Our recommendations regarding simultaneous surgery for bilateral multiple pulmonary nodules include the following: (1) Evaluate the surgical indications and the extent of resection for each individual nodule prior to surgery according to 3D reconstruction of the nodules. (2) The scope of resection should include no more than ten segments during the same period. If bilateral lobectomy is needed, staging surgery is recommended, except for the right middle lung, and sequential surgery is not recommended if one lung requires combined lobectomy, sleeve lobectomy or even pneumonectomy. Sublobectomy (wedge or segmentectomy) can be performed for cases of microinvasion and primary adenocarcinoma. The precancerous lesions should be followed up regularly or in cases of ipsilateral wedge resection because of the small total resection range. (3) The priority side of the operation is the side requiring less invasive resection, such as wedge resection before segmentectomy and segmentectomy before lobectomy. If the bilateral excision areas are equal, the right-side operation should be performed first. When there is a small amount of pneumothorax, the pneumothorax side should be operated on first to prevent further aggravation during surgery. (4) A comprehensive diagnosis should be made according to the frozen results in combination with consideration for the size of the lesion, the proportion of solid components and imaging findings, and the mode of operation should be adjusted if necessary.

Two-stage surgery for bilateral pulmonary nodules is relatively safe, but some problems remain, such as the time and economic and psychological burdens placed on the patient. one-stage surgery also has some problems, such as causing relatively greater trauma. Studies have shown that it is safe to comprehensively evaluate the preoperative imaging findings and cardiopulmonary function reserve so that appropriate patients can be selected and a reasonable mode of operation can be developed for bilateral simultaneous thoracoscopic surgery (24, 25). In this study, the one-stage group had less pleural drainage (375.95 ± 243.69 ml), a shorter chest tube indwelling duration (3.38 ± 1.49 days) and shorter postoperative hospitalization duration (4.08 ± 1.81 days) than the two-stage group (P<0.05). No serious complications or serious complications occurred during the perioperative period. Two patients had postoperative pneumonia and were discharged after anti-inflammatory treatment. Three patients had transient atrial fibrillation that improved after symptomatic treatment and did not recur during follow-up. One patient had persistent severe air leakage, which was confirmed to be caused by rupture of the pulmonary bullae, and this patient was successfully discharged after surgical resection of the pulmonary bullae. One patient who underwent segmentomy-lobectomy experienced haemoptysis and was discharged after anti-inflammatory and haemostatic treatment. The results of this study confirmed that one-stage VATS is safe and feasible. Our experience is that the following should be taken into account to ensure surgical success: (1) Strict grasp of indications: A good evaluation of cardiopulmonary function is needed to avoid selecting elderly individuals or patients with underlying cardiopulmonary diseases. (2) Adequate preoperative evaluation: Thin-slice chest CT and 3D reconstruction should be performed routinely before the operation, a 3D reconstruction model can be used for accurate localization of nonperipheral pulmonary nodules without wedge resection, and a disposable pulmonary nodule puncture needle can allow for preoperative localization of nonsubpleural nodules. (3) To establish a reasonable mode of operation, pneumonectomy, combined lobectomy and sleeve resection should be avoided on the side of the main nodule, and staged surgery is recommended if the abovementioned operation is necessary after evaluation. With the exception of the right middle lung, simultaneous lobectomy is not recommended. (4) Fine operation capability during surgery: accurate segmentomy and subsegmentomy with fluorescence endoscopy can quickly display the intersegmental boundary and obviously shorten the operation time. If there is air leakage at the surgical stump, such as the complex lung segment, a broken thread and suture stump can be used to reduce the occurrence of pulmonary air leakage by spraying the wound with protein adhesive. For wedge resection and no air leakage, a thin flexible tube negative pressure ball can be retained, which can effectively reduce postoperative pain. (5) Rapid rehabilitation during the perioperative period: Pulmonary function exercises should be performed before and after the operation, and respiratory tract management is needed after the operation, along with a guided diet to improve nutrition; early ambulation and good pain management can help to prevent complications and promote rehabilitation.

MPLCs refer to two or more primary lung cancers detected at the same time or found in succession in a single patient. In 1898, Billroth first reported the case of a patient with MPLC (26). In 1924, Beyreuther first described the concept of multiple primary cancers (27). In 1975, Martini and Melamed described the first diagnostic criterion for MPLC (8). At present, the underlying mechanism of MPLCs remains unclear; some scholars believe that it is caused by multisite gene mutations (28). Seventy-five percent of the patients in this study were confirmed to have SMPLCs, and most of them were stage IA or earlier. Although there is no consensus on the treatment of bilateral SMPLCs, some studies have shown that the radical resection of bilateral lesions may be the optimal treatment choice and could confer a good long-term prognosis (29, 30). We offer the following suggestions: (1) A multidisciplinary team (multidisciplinary team, MDT) consultation should be performed to synthesize the opinions of various disciplines so that the most reasonable treatment plan can be provided to the patient. (2) For early MPLCs, priority should be given to surgical treatment, and suitable patients should choose bilateral uniportal VATS. (3) For patients who cannot undergo bilateral surgery, clinicians should first treat the main lesions and then perform staged surgery after an interval of 1-6 months. (4) Lesions that are not resected tend to become malignant during follow-up. Radiofrequency ablation or stereotactic radiotherapy can be considered if the patient cannot undergo a repeat operation after evaluation.

There were some limitations to our study. First, this study was a single-center analysis, so there was a certain degree of selection bias. Second, the sample size was relatively small due to objective reasons. Third, the follow-up time was short, and some patients in this study were unavailable for follow-up. Finally, this study was limited to mutual learning and discussion among peers, which is related to the professional foundation, clinical experience, surgical proficiency, and tacit cooperation of the surgical team, among other factors. Prospective studies with a larger number of patients and longer follow-up time are necessary.

## Conclusions

Bilateral one-stage thoracoscopic lung surgery is safe and feasible for the treatment of bilateral multiple primary lung cancers. Strict control of the surgical indications, reasonable preoperative planning, accurate intraoperative surgery, and standardized perioperative management can effectively reduce complications and maximize benefits in suitable patients.

## Data availability statement

The original contributions presented in the study are included in the article/supplementary material. Further inquiries can be directed to the corresponding authors.

## Author contributions

GX and GW, conceptualization, methodology, software, investigation, writing - original draft, writing - review and editing. XM and MW, visualization and investigation. TL and MX, funding acquisition, writing - review and editing.

## Funding

This work was supported by the grants from the National Natural Science Foundation of China and Key research and development projects in Anhui Province (NO.81973643 and 202004j07020017).

## Acknowledgments

The authors would like to thank the Department of Thoracic Surgery of the First Affiliated Hospital of USTC for supporting this study. No external funding source was involved.

## Conflict of interest

The authors declare that the research was conducted in the absence of any commercial or financial relationships that could be construed as a potential conflict of interest.

## Publisher’s note

All claims expressed in this article are solely those of the authors and do not necessarily represent those of their affiliated organizations, or those of the publisher, the editors and the reviewers. Any product that may be evaluated in this article, or claim that may be made by its manufacturer, is not guaranteed or endorsed by the publisher.
